# The Effect of Neuromodulatory Drugs on the Intensity of Chronic Pelvic Pain in Women: A Systematic Review

**DOI:** 10.1055/s-0042-1755459

**Published:** 2022-08-31

**Authors:** Marcela Almeida Andrade, Leila Cristina Soares, Marco Aurélio Pinho de Oliveira

**Affiliations:** 1 Universidade do Estado do Rio de Janeiro, Rio de Janeiro, RJ, Brazil

**Keywords:** chronic pelvic pain, management, neuromodulations drugs, gabapentin, sertraline, dor pélvica crônica, conduta, drogas neuromoduladoras, gabapentina, sertralina

## Abstract

**Objective:** To evaluate the effect of neuromodulatory drugs on the intensity of chronic pelvic pain (CPP) in women.

**Data sources:** Searches were carried out in the PubMed, Cochrane Central, Embase, Lilacs, OpenGrey, and Clinical Trials databases.

**Selection of studies:** The searches were carried out by two of the authors, not delimiting publication date or original language. The following descriptors were used: *chronic pelvic pain in women* OR *endometriosis* , associated with MESH/ENTREE/DeCS: *gabapentinoids* , *gabapentin* , *amitriptyline* , *antidepressant* , *pregabalin* , *anticonvulsant* , *sertraline* , *duloxetine* , *nortriptyline* , *citalopram* , *imipramine* , *venlafaxine* , *neuromodulation drugs* , *acyclic pelvic pain* , *serotonin* , *noradrenaline reuptake inhibitors* , and *tricyclic antidepressants* , with the Boolean operator *OR* . Case reports and systematic reviews were excluded.

**Data collection:** The following data were extracted: author, year of publication, setting, type of study, sample size, intervention details, follow-up time, and results.

**Data synthesis:** A total of 218 articles were found, with 79 being excluded because they were repeated, leaving 139 articles for analysis: 90 were excluded in the analysis of the titles, 37 after reading the abstract, and 4 after reading the articles in full, and 1 could not be found, therefore, leaving 7 articles that were included in the review.

**Conclusion:** Most of the studies analyzed have shown pain improvement with the help of neuromodulators for chronic pain. However, no improvement was found in the study with the highest statistical power. There is still not enough evidence that neuromodulatory drugs reduce the intensity of pain in women with CPP.

## Resumo

**Objetivo:** Avaliar o efeito de drogas neuromoduladoras na intensidade da dor pélvica crônica em mulheres.

**Fontes de dados:** As buscas foram realizadas nas bases de dados PubMed, Cochrane Central, Embase, Lilacs, OpenGrey e Clinical Trials.

**Seleção dos estudos:** As buscas foram realizadas por dois dos autores, não delimitando data de publicação ou idioma de publicação. Foram usados os seguintes descritores: *chronic pelvic pain in women* OR *endometriosis* , associated with MESH/ENTREE/DeCS: *gabapentinoids* , *gabapentin* , *amitriptyline* , *antidepressant* , *pregabalin* , *anticonvulsant* , *sertraline* , *duloxetine* , *nortriptyline* , *citalopram* , *imipramine* , *venlafaxine* , *neuromodulation drugs* , *acyclic pelvic pain* , *serotonin* , *noradrenaline reuptake inhibitors* e *tricyclic antidepressants* , com o operador booleano *OR* . Relatos de caso e revisões sistemáticas foram excluídos.

**Coleta de dados:** Foram extraídos os seguintes dados: autor, ano de publicação, local de origem, tipo de estudo, tamanho da amostra, detalhes da intervenção, tempo de seguimento e resultados.

**Síntese dos dados:** Foram encontrados 218 artigos, sendo 79 deles excluídos por serem repetidos, restando 139 artigos para análise, dos quais 90 foram excluídos na análise dos títulos, 37 após a leitura do resumo e 4 após a leitura dos artigos na íntegra, e 1 não foi encontrado, restando, então, 7 artigos que foram incluídos na revisão.

**Conclusão:** A maioria dos estudos analisados mostrou melhora da dor crônica com auxílio de neuromoduladores. No entanto, nenhuma melhora foi encontrada no artigo com maior poder estatístico. Ainda não há evidências suficientes de que drogas neuromoduladoras reduzam a intensidade da dor pélvica crônica em mulheres.

## Introduction

Chronic pelvic pain (CPP) is a frequent complaint in primary care, present in 2.1 to 24% of the world's female population, and is responsible for 20% of gynecological consultations. 1 This condition greatly impacts the patient's quality of life, interfering with sexual activity, urination, and work activities, among others, thus representing a major socio-sanitary problem. 2

The frequent inexistence of a causal link between examination findings and clinical reality means that CPP treatment does not always bring satisfactory results. Pelvic pain can be of gynecological, urological, gastrointestinal, musculoskeletal, or psychiatric causes, and it usually has a visceral or neuropathic origin. Neuropathic pain can be induced by various pathological situations, all of them altering the physiology of the nervous system. 3

Among patients with CPP, it is estimated that one-third are caused by endometriosis, the most common diagnosis in this population. Usually of multifactorial origin, the pathophysiology of CPP suggests that there is a final common share of inflammatory and neurogenic insults, which finally manifest as chronic pain. Somatic pain thresholds for different stimuli are lowered in painful and nonpainful locations in CPP, indicating generalized hyperalgesia (increased sensitivity to pain) and central sensitization. 1 4 5

There are no standard treatments for visceral and neuropathic pain. Central sensitization plays an important role in the development and maintenance of neuropathic pain symptoms. 6 When neuropathic pain is diagnosed, neuromodulatory drugs are usually prescribed, and the first-line drugs recommended include tricyclic antidepressants, such as amitriptyline, nortriptyline selective serotonin-noradrenaline reuptake inhibitors (duloxetine), and anticonvulsants, such as gabapentin, gabapentinoid, and pregabalin, but side effects often limit their clinical use. 3 6

The analgesic activity of tricyclic antidepressant agents remains questionable. 7 Gabapentin primarily affects pain modulation by the central neural system, and gabapentinoid drugs affect brain function in models of central sensitization and in patients with chronic pain. 8

Although neuromodulatory drugs are already used empirically in the treatment of women with CPP, there is still no robust scientific evidence for their use in this group of patients. There is only one review paper on this topic, from 1993, at a time when knowledge regarding these drugs in CPP was still limited. 9

This study aims to evaluate the effect of neuromodulatory drugs on the intensity of CPP in women.

## Methods

A protocol was registered with PROSPERO with the number ID: CRD42020171938. The review was carried out following the Preferred Reporting Items for Systematic Reviews and Meta-Analyses (PRISMA) recommendations (Main Items for Reporting Systematic Reviews and Meta-analyses). 10

Searches were made in the PubMed, EMBASE, and Lilacs databases, accepting studies from 1966 to May 2020, as well as the Cochrane Central and Clinical Trials, with studies from 1966 to May 2020. A search for gray literature was also carried out in the OpenGrey database, Google Scholar, and WorldCat, for studies published between January 2010 and May 2020. Due to the reduced number of studies on women in this area, this work chose to be as comprehensive as possible, not delimiting publication date, published language, number of patients, among others. The searches were carried out independently by the two authors of the review. We also included *endometriosis* in the search strategy since it is considered one of the main causes of CPP, being widely studied.

Neuromodulatory drugs have also been described specifically according to those available to treat other causes of chronic pain. The following descriptors were used: *chronic pelvic pain in women* OR *endometriosis* , associated with MESH/ENTREE/DeCS: *gabapentinoids* , *gabapentin* , *amitriptyline* , *antidepressant* , *pregabalin* , *anticonvulsant* , *sertraline* , *duloxetine* , *nortriptyline* , *citalopram* , *imipramine* , *venlafaxine* , *neuromodulation drugs* , *acyclic pelvic pain* , *serotonin* , *noradrenaline reuptake inhibitors* , and *tricyclic antidepressants* , with the Boolean operator *OR* .

The eligibility criteria for inclusion of the study in the systematic review established were: female patients; CPP, defined here as pain in the pelvic region lasting more than 3 months, not considering vaginismus and interstitial cystitis because they are well-studied; treatment of pain with neuromodulatory medication (antidepressants and anticonvulsants); and studies whose objective is to assess the improvement of CPP after the use of neuromodulatory drugs. Case reports and systematic reviews were excluded.

The search performed in the gray literature was conducted using the same principles, words, and MeSHs as the search for published articles.

When reading the full text of each article identified for inclusion in the review as part of the data extraction process, scales for evaluating the quality of each selected study were applied.

The questionnaire obtained from the Centre for Evidence-Based Medicine's (CEBM) critical appraisal tools, from Oxford University, was used to evaluate quality. All studies went through quality control and proceeded to the data extraction phase. Only case reports and systematic reviews were excluded. A form was drawn up to extract data from the selected studies. The specific characteristics of each study were recorded. Data extracted from each study included the first author, year of publication, country of origin, type of study, sample size, intervention details, follow-up time, and results.

## Results

**Fig. 1:**
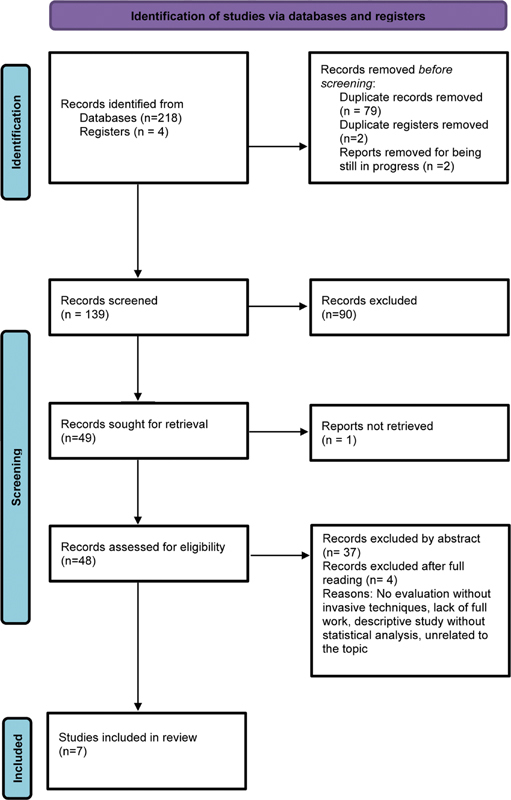
Flow diagram of selection and eligibility of studies.

A total of 218 articles were initially found. After removing 79 duplicates, 90 articles were excluded by title, 37 by abstract, and 4 after full reading; furthermore, 1 article could not be found ( Fig. 1 ). No extra works were found through OpenGrey, Google Scholar, and Worldcat. Through ClinicalTrials, only 4 studies were found, and 2 of them were already included in the search list of published articles (at Cochrane Central). The other 2 studies 11 12 are still in progress and are unrelated to the review. Therefore, it was decided not to include the findings from the gray literature in this work. None of the selected studies was directed to a specific pelvic pathology, and, in 3 of them, concomitant pelvic pathology was an exclusion criterion.

## No Difference in Pelvic Pain Control with Neuromodulatory Drugs

Of the 3studies that found no significant difference in pain control, one tested the effects of sertraline and two of gabapentin. In 1998, Engel et al. 13 conducted a study to evaluate the effects of sertraline as a treatment for CPP in 23 women with persistent pelvic pain for more than or equal to 3 months. This study was a double-blind, randomized, placebo-controlled, crossover trial. The variables of interest were assessed before treatment and 6 weeks after starting the medication. After 2 weeks without medication (washout), the two types of treatment were crossed (crossover), and new measures were taken before and after 6 weeks. The pain was assessed using the composite pain intensity (CPI) score. Although the Structured Clinical Interview Guide for the Hamilton Rating Scale for Depression, Hamilton Rating Scale for Depression (HAM-D), and CPI scores were, on average, slightly better with sertraline than with placebo, the results were not statistically significant. However, it caused a noticeable, statistically significant decrease in the SF-36's Role Functioning – emotional scale in the sertraline group. 13

Seretny et al. 14 published a randomized, controlled trial in 2019, with 12 patients, in which the authors concluded that the gabapentin group did not have significant difference from the placebo group in pain control after 3 months.

In 2020, Horne et al. 8 conducted a double-blind, placebo-controlled randomized trial with 306 participants, and found that treatment with gabapentin did not result in significantly lower pain scores in women with CPP, and was associated with higher rates of side effects than placebo. The pain was assessed using the numerical rating scale (NRS). As the participants were able to remain on any analgesics they were taking, the authors did not exclude women who realized they were taking placebo and compensated by increasing the use of analgesics, leading to a smaller difference in the effect of gabapentin. This is the study with the largest sample size among all. 8

## Improvement in Pelvic Pain Control with Neuromodulatory Drugs

Of the 4 studies that found improvement in pelvic pain control, 1 tested the effect of nortriptyline, 2 tested gabapentin, and 1 compared gabapentin, amitriptyline, and gabapentin with amitriptyline. In a 1991 study, Walker et al. 15 evaluated the use of nortriptyline in the treatment of women with CPP. The studied population was composed of women with CPP for a period equal to or greater than 6 months, having obtained a total initial sample size of 14 women, of whom 7 completed the study, which may hinder more robust conclusions. The study was a clinical trial (with pretreatment evaluations, at 1 and 2 months after the beginning of the treatment), and the pain was assessed using the Visual Analogue Scale (VAS). The pain was associated with the diagnosis of depression in 4 of the 7 patients (57%) who completed the study. In the 2-month evaluation, 6 of the 7 women (83%) who remained on treatment were pain-free or reported that their pain was significantly less on the HAM-D. However, this is a low-impact study due to its sample size and design. 15

Sator-Katzenschlager et al., 6 in a 2005 study, compared the efficacy and side effects of gabapentin, amitriptyline, and their combination in women with CPP. Of the 56 patients studied, subdivided into 3 treatment groups, 20 received gabapentin (n=20), 20 received amitriptyline (n=20), and another 16 received a combination of both drugs (n=16). The study was conducted as an open, prospective, randomized clinical trial (2-year follow-up). The VAS score for pain intensity was measured at pre-treatment (0) and at 1, 3, 12, and 24 months after starting the treatment. Pain intensity (VAS) before treatment was similar between groups, and all patients had significant pain relief at all times investigated compared to the pain score before treatment. However, after 6, 12, and 24 months, pain relief was significantly better in patients who received gabapentin alone or combined with amitriptyline, when compared with patients who received only amitriptyline. It is noteworthy that there was no statistically significant difference in pain relief between the gabapentin only group and the gabapentin in combination with amitriptyline group. The incidence of side effects was lower in the gabapentin group than in the other two that included amitriptyline. 6

In 2016, Lewis et al. 16 conducted a study with the primary objective of determining the recruitment and retention levels of study participants, and a secondary objective of estimating the potential efficacy of gabapentin in women with CPP. Of the 47 women who started the study, only 25 remained until the end. The pilot study used several instruments, including the Visual Analog Scale (VAS), Brief Pain Inventory (BPI), Pain Disability Questionnaire (PDQ), Hospital Anxiety and Depression Score (HADS), Quality of Life EQ5D (QOL-EQ5D), World Health Organization's (WHO) QOL, and the Medical Outcome Profile (MYMOP). The gabapentin group responded significantly better to pain management (use of BPI) than participants in the placebo group (difference of 1.72 points, 95% confidence interval [CI]: 0.07–3.36) at 6 months. Additionally, the HADS analysis revealed an improvement in mood in the gabapentin group (4.35 points, 95% CI: 1.97–6.73) at 6 months. Of the 22 women in the gabapentin group, 17 (77%) had at least one adverse event, when compared with 16 of the 25 (64%) in the placebo group. Most of these events were mild (15 in each group). There were two serious adverse events reported, both in the gabapentin group. However, both were exacerbations of CPP involving hospitalization, probably not related to an adverse effect of gabapentin. 16

Another study was conducted in 2019 by AbdelHafeez et al., 17 a randomized, double-blind clinical trial whose main objective was to evaluate the efficacy of oral gabapentin in pain relief in women with idiopathic CPP. Measurements were performed in the initial phase (pretreatment), then at 12 and 24 weeks after starting the treatment. The initial sample size included 60 women, and after 12 weeks, 50 women remained. Only 34 women remained at 24 weeks (end of the study). The outcomes of interest were pain intensity (VAS), general patient satisfaction, and adverse effects of gabapentin. The pain score was significantly lower in the gabapentin group compared with the placebo group after 12 and 24 weeks. A significant reduction in pain was found at 24 weeks compared with pretreatment in the gabapentin group, which was not detected in the placebo group. At 24 weeks, a significantly higher proportion of patients reported a 30% or greater reduction in pain scores: 19 out of 20 patients (95%) in the gabapentin group, compared with 5 out of 14 patients (35.7%) in the placebo group. A significantly higher incidence of dizziness was found in the gabapentin group compared with the placebo group ( Chart 1 ). The dropout rate was 33.3% (10) in the group gabapentin, compared with 53.3% (16) in the placebo group. The authors concluded that CPP in women could be treated sufficiently, though not completely, with gabapentin. 17

**Chart 1:** Selected studies with author/year of publication/country of origin, study design, population/sample size, objectives, assessment methods, follow-up time, results, and quality of evidence

Author/year/ setting	Study design	Sample size	Intervention details	Pain measurement	Follow-up	Results	Quality of evidence
Walker et al. (1991) 15 United States	Longitudinal (no control group)	14	Nortriptyline 100mg/d after a 2-week upward titration.	VAS	2 months	6 of the 7 women (83%) who completed the treatment were pain free or reported that their pain was significantly less ( *p* <0.03).	LOW No comparison groups; high dropout rate (7patients were lost due to side effects).
Engel et al. (1998) 13 United States	Randomized double-blind, placebo-controlled, crossover trial	23	Randomization in two groups: 50mg/d bid of sertraline and placebo. After two weeks without medication (washout), the two groups were crossed, and evaluated after six weeks.	Composite Pain Intensity (including Brief Pain Inventory)	6 weeks	The authors were unable to conclude that sertraline had a significant analgesic effect compared to placebo in women with CPP.	LOW No selection, attrition, or confounding bias. Small sample size (underpowered to detect medium effect sizes).
Sator-Katzenschlager et al. (2005) 6 Austria	Randomized, controlled trial	56	20 received gabapentin – 300mg/d until VAS 3 (n=20), 20 received amitriptyline – 25mg/d until VAS 3 (n=20), and 16 received – 300mg gabapentin+25mg amitriptyline/d until VAS 3 (n=16).	VAS	24 months	After 6, 12, and 24 months, pain relief was significantly better in patients who received gabapentin alone or in combination with amitriptyline than in patients who received only amitriptyline.	LOW 7 patients were lost in follow-up. High attrition bias. Randomization sequence unclear
Lewis et al. (2016) 16 United Kingdom	Randomized controlled trial	47	22 gabapentin and 25 placebo. 300mg gabapentin/d (with increased in 300mg/d increments each week until they reported a 50% pain reduction or side effects)	Brief Pain Inventory	6 months	The gabapentin group responded significantly better to pain management than participants in the placebo group (difference of 1.72 points, 95% CI: 0.07–3.36) at 6 months.	LOW Possibility of attrition bias – of the 47 women who started the study, but only 25 remained to the end.
Seretny et al. (2019) 14 United Kingdom	Randomized, controlled, pilot study	12	6 gabapentin and 6 placebo. 300mg gabapentin/d (with increased in 300mg/d increments each week until they reported a 50% pain reduction or side effects).	VAS	12 weeks	After 3 months, the mean difference between groups were non-significative 0.80 (-1.12, 2.72).	LOW No selection, attrition, or confounding bias. Small sample size (underpowered to detect medium effect sizes).
Abdelhafeez et al. (2019) 17 Egypt	Randomized, double-blinded, clinical trial	60	Women were divided into two equal groups: placebo and gabapentin 900mg (with 300mg weekly incremental dose until the pain was controlled or severe side effects occurred, or a maximum daily dose of 2700mg was reached).	VAS	6 months	At week 24, there was a significantly higher proportion of patients reporting a 30% or greater reduction in pain scores: 19 out of 20 patients (95%) in the gabapentin group, compared to 5 out of 14 patients (35.7%) in the placebo group.	MODERATE After 12 weeks, there were 50 women. No selection, attrition, or confounding bias.
Horne et al. (2020) 8 United Kingdom	Randomized, double-blind, placebo-controlled trial	306	Participants were randomly assigned in a 1:1 ratio to receive gabapentin (titrated to a maximum dose of 2700mg daily) or placebo.	Brief Pain Inventory	13–16 weeks	Treatment with gabapentin did not result in significantly lower pain scores in women with CPP and was associated with higher rates of side effects than placebo.	HIGH Adequate sample size. No selection, attrition, or confounding bias

**Abbreviation:** CPP, chronic pelvic pain; VAS, visual analogue scale.

In relation to the quality of the selected studies that used gabapentin, we found no selection, attrition, confounding, or outcome bias in the studies by Horne et al., 8 Sator-Katzenschlager et al., 6 and Abdelhafeez et al. 17 In Lewis et al.'s study, 16 an attrition bias was observed. In the study of Walker et al., 9 the only one that studied the effect of nortriptyline in CPP, there was no control group, a low sample size, and a high dropout rate, showing the low quality of this study. In the study of Engel et al., 13 the only that studied the effect of sertraline in CPP, there were selection and confounding biases, but no attrition bias was observed. They did not perform sample size calculation or multivariate analysis, showing an overall low quality of this study.

## Discussion

The use of neuromodulatory drugs in the treatment of CPP in women, despite being widely used in clinical practice, lacks more robust scientific evidence. The number of studies included in this systematic review corroborates this statement. After extensive research in the literature, only 7 studies were eligible, all of them with small sampling, and 2 of them published more than 20 years ago. 9 13 15 The findings of the 2 more recent studies 17 show that better understanding of the effectiveness of treatment in this group of women is becoming necessary, as well as supporting the clinical practice already employed by physicians in an empirical way.

Studies related to chronic pain 18 19 20 have already shown that selective serotonin receptor inhibitors are not effective in improving chronic pain, a finding corroborated by Engel et al.'s study. 13 Despite the small sample size, the authors suggest not overestimating the use of sertraline in the treatment of CPP in women, since it was not possible to find a statistically significant improvement in pain with treatment.

Tricyclic antidepressants are the neuromodulator drugs that have been studied the longest in the treatment of chronic pain, with proven efficacy in several causes of pain, but many side effects. Walker et al.'s 15 study showed that the use of nortriptyline in women with CPP appears to be effective, a fact that is validated by the effectiveness of nortriptyline in other causes of chronic pain. However, 7 of the 14 women who did not complete the study had a higher frequency of side effects, when compared to the group that completed the study. Due to the small sample size and methodological vulnerability, we should only consider it as a preliminary study. 15

More recent studies have evaluated the use of other drugs in search of an equal or better response to antidepressants, but with lesser side effects. Dual serotonin and norepinephrine reuptake inhibitors (SNRI) and antiepileptics such as gabapentin and pregabalin, have been gaining more and more space in these studies. 21 22 23

The study conducted by Sator-Katzenschlager et al., in 2005, 6 aimed to compare the effectiveness in improving CPP using amitriptyline associated with gabapentin, and each one alone. The study was able to conclude that all groups showed significant pain relief when compared with pretreatment scores. Another important fact highlighted in the study was that the incidence of side effects was lower in the gabapentin group alone when compared with the use of amitriptyline alone or in conjunction with gabapentin. These findings suggest that gabapentin is as effective alone or combined with amitriptyline in relieving CPP in women, with fewer side effects and, probably, better treatment adherence. 6

Still on the use of gabapentin, 2 recent studies evaluated its use compared to placebo in the treatment of CPP. The results showed that gabapentin could be considered an alternative treatment line for cases of CPP in women, which is not relieved by painkillers. The pilot study conducted by Lewis et al. 16 evaluated the improvement in CPP using gabapentin. The study started with 47 patients, with losses during its conduction, and was completed with a final sample of 25 patients. The study was able to show that gabapentin was significantly better than placebo in improving pain and anxiety, without, however, indicating a higher incidence of side effects. A higher proportion of women in the gabapentin group had a severe adverse event than in the placebo group, and this was an important cause of treatment dropout. The authors concluded that the pilot study supports the feasibility of a future large, randomized, controlled, multicenter study to determine the effectiveness of gabapentin in the treatment of CPP. 16 Such study has already started in 2018, being now in the recruitment phase, and intends to evaluate 300 patients so as to bring more concrete answers regarding the use of gabapentin for treatment of CPP in women. 24

In 2019, Seretny et al. 14 showed no effect with the use of gabapentin for pain reduction. However, it was a study with a short follow-up time and small number of participants. Meanwhile, in the same year, AbdelHafeez et al., 17 in a study that started with 60 patients and had a final sample of 34, showed that the pain score in the gabapentin group was significantly reduced when compared with the placebo group at 12 and 24 weeks. The side effects were slightly higher in the gabapentin group, but with a higher dropout rate in the placebo group, justified by most patients as due to the lack of pain improvement. 17

During this review, we observed a lack of studies on other drugs, such as pregabalin, that demonstrate an effect on chronic pain relief at other sites. There are studies comparing its effectiveness with that of gabapentin in the treatment of chronic pain in general, showing that both are effective in reducing the pain score, with pregabalin showing a higher incidence of side effects when compared to gabapentin. 11 25 A study by Agarwal et al. 12 compared the efficacy of gabapentin and pregabalin in men with chronic urological pelvic pain, and concluded that gabapentin was significantly more effective in controlling pain when compared to pregabalin. Of the 54 patients who received pregabalin in this study, 20 needed to switch to gabapentin due to lack of improvement in the pain score, and 24 needed to associate the use of amitriptyline with pregabalin to achieve pain control. 12

A 2017 review by Senderovich et al. 26 assessed the combined use of pregabalin and gabapentin in pain control. Despite their pharmacokinetic similarities, they have been used together in clinical and research situations, and have been found to have a synergistic effect on pain control. The authors concluded that pharmacokinetics, drug interactions, and adverse reactions to this combination should be considered before combined therapy with gabapentin and pregabalin is proposed as first-line treatment in situations of refractory pain and in patients with low levels of tolerance to an agent alone. It is important to note that only the most recent study by Horne et al. (2020), 8 had a considerable number of samples with few losses, and, in that study, there was no demonstration of the superiority of gabapentin compared with placebo. 8 Other studies with large samples and long follow-up periods should be carried out to determine the effectiveness of neuromodulatory drugs in the control of CPP.

One of the main limitations of this systematic review is the small number of articles found; furthermore, of the many potential neuromodulatory drugs for neuropathic pain, there are only a few used for the treatment of CPP in women. The most studied drug is gabapentin, and we did not find studies using pregabalin. Additionally, the doses of neuromodulators are not standardized and, despite being similar between studies, it prevents a more accurate comparison. Also, the overall quality of the studies is not satisfactory, with few randomized studies of good quality, which makes it difficult to reach a conclusion on the final recommendation for the use or not of neuromodulatory drugs in the treatment of CPP.

## Conclusion

Most of the studies analyzed have shown pain improvement with the help of neuromodulator drugs for chronic pain. However, the most powerful and high-quality study did not show pain improvement. In the study with the longest follow-up time, pain relief was significantly better in patients who received gabapentin alone or combined with amitriptyline than in patients who received only amitriptyline. It is possible that the high loss of participants to follow-up could be due to frequent side effects and lack of immediate perceived response. Furthermore, we did not find studies specifically with women with endometriosis and CPP; therefore, these results cannot be extended to this common cause of CPP. We conclude that there is no robust evidence to either indicate or avoid the use of neuromodulatory drugs in CPP, and further high-quality studies, especially randomized controlled trials, are needed to support the use of these drugs in reducing the intensity of pain in women with CPP.
